# *mSphere* Highlights Black In Microbiology Week

**DOI:** 10.1128/mSphere.00966-20

**Published:** 2020-09-30

**Authors:** Michael D. L. Johnson

**Affiliations:** a Department of Immunobiology, University of Arizona, Tucson, Arizona, USA; b BIO5 Institute, University of Arizona, Tucson, Arizona, USA; c Valley Fever Center for Excellence, University of Arizona, Tucson, Arizona, USA

## Abstract

The inaugural Black In Microbiology Week (#BlackInMicro) is 28 September 2020 through 4 October 2020. Its mission is to “showcase the presence and accomplishments of Black microbiologists from around the globe, connect Black microbiologists with one another and foster a sense of community among them, and provide a forum for the discussion of racial disparities in microbiology and its subfields.” Participation in this event will happen primarily over Twitter through the hashtag #BlackInMicro and over Zoom through registration on the website https://blackinmicrobiology.org/. An additional mission of Black In Microbiology Week is to amplify black scientists. Today, *mSphere* does this by presenting two mSphere of Influence commentaries from Black In Microbiology co-lead organizers Ariangela J. Kozik (“mSphere of Influence: frameshift—a vision for human microbiome research” [mSphere 5:e00944-20, 2020, https://doi.org/10.1128/mSphere.00944-20]) and Kishana Taylor (“mSphere of Influence: that’s racist—COVID-19, biological determinism, and the limits of hypotheses” [mSphere 5:e00945-20, 2020, https://doi.org/10.1128/mSphere.00945-20]).

## Abstract (precis)

The inaugural Black In Microbiology Week (#BlackInMicro) is 28 September 2020 through 4 October 2020. Its mission is to “showcase the presence and accomplishments of Black microbiologists from around the globe, connect Black microbiologists with one another and foster a sense of community among them, and provide a forum for the discussion of racial disparities in microbiology and its subfields.” Participation in this event will happen primarily over Twitter through the hashtag #BlackInMicro and over Zoom through registration on the website https://blackinmicrobiology.org/.

## EDITORIAL

Whether the idea is projected internally or externally, eliminating the perception of a person being inferior based on race or gender is a difficult, uphill climb. Lack of representation and visibility in a given area impede the ability to progress. While there have been many statements in solidarity with the Black Lives Matter movement and against the plague of racism, unless action is taken based on the words of wisdom provided, the needle of change will stagnate. Lip service is as the wasp that uses surface tension to drink water from the pond, delicately balancing on the tiny ripples caused by the wind, not causing new ones, and flying away when done, while action, and what is needed, is the stone that is skipped to spread the message through its ripples or that is dropped in to cause a big splash. Dare to be the stone, the rock that can be relied on, not the wasp that will fly away only to return when it needs to feel nourished.

While taking action might be an intimidating first step, we must be bold in taking it. There is no shortcut. For this reason, we as a journal and a scientific society not only support the Black Lives Matter movement with our words but we also support it with our actions of amplification and allyship (1). #BlackInMicro Week, which celebrates being Black in microbiology, starts 28 September 2020 and goes through 4 October 2020. While this week highlights representation, accomplishments, disparities, community, and stories of being Black in microbiology, the expectation and hope is that the ripples continue long afterwards.

We, as *mSphere*, highlight the two co-lead organizers of Black In Microbiology, Drs. Ariangela J. Kozik and Kishana Taylor, in giving them the opportunity to share what science has impacted their career by having them each write an mSphere of Influence commentary. Dr. Kozik in her article entitled “mSphere of Influence: frameshift—a vision for human microbiome research” discusses microbiome diversity and its relationship to disease disparities (2), and Dr. Taylor in her article entitled “That’s racist—COVID-19, biological determinism, and the limits of hypotheses” rightfully attacks the bias behind many hypotheses of health disparities research (3). Additionally, the American Society for Microbiology is also committing financial sponsorship for both this week and for annual programming, professional development content, and their online platforms to promote Black In Microbiology Week. While these commentaries and some of the issues discussed in #BlackInMicro Week (and beyond) might make you feel uncomfortable, your challenge will be to lean in, listen, learn, stand with this community, and dare to be the stone.

## References

[1] Schloss PD, Junior M, Alvania R, Arias CA, Baumler A, Casadevall A, Detweiler C, Drake H, Gilbert J, Imperiale MJ, Lovett S, Maloy S, McAdam AJ, Newton ILG, Sadowsky MJ, Sandri-Goldin RM, Silhavy TJ, Tontonoz P, Young JH, Cameron CE, Cann I, Fuller AO, Kozik AJ. 2020. The ASM Journals Committee values the contributions of Black microbiologists. mSphere 5:e00719-20. doi:10.1128/mSphere.00719-20. 32737154 PMC7398635

[2] Kozik AJ. 2020. mSphere of Influence: frameshift—a vision for human microbiome research. mSphere 5:e00944-20. doi:10.1128/mSphere.00944-20. 33025908 PMC7529440

[3] Taylor K. 2020. mSphere of Influence: that’s racist—COVID-19, biological determinism, and the limits of hypotheses. mSphere 5:e00945-20. doi:10.1128/mSphere.00945-20. 33025909 PMC7529441

